# Examination of sex and minocycline treatment on acute morphine-induced analgesia and inflammatory gene expression along the pain pathway in Sprague–Dawley rats

**DOI:** 10.1186/s13293-015-0049-3

**Published:** 2015-12-12

**Authors:** Caitlin K. Posillico, Laurne S. Terasaki, Staci D. Bilbo, Jaclyn M. Schwarz

**Affiliations:** Department of Psychological and Brain Sciences, University of Delaware, 108 Wolf Hall, Newark, DE 19716 USA; Department of Psychology and Neuroscience, Duke University, 572 Research Dr., Durham, NC 27708 USA

**Keywords:** Morphine, Microglia, Astrocytes, Minocycline, Sex, Analgesia

## Abstract

**Background:**

In addition to its classical effects on opioid receptors, morphine can activate glia and stimulate the production of pro-inflammatory immune molecules which in turn counteract the analgesic properties of morphine. We hypothesized that decreased morphine analgesia in females may be the result of exaggerated microglial activation in brain regions critical for analgesia.

**Methods:**

Male and female rats were treated with morphine and/or minocycline and morphine analgesia was examined using the hot plate. We also examined the expression of microglial and astrocyte markers in the pain pathway.

**Results:**

Males treated with minocycline, a microglial inhibitor, exhibited a significant increase in acute morphine analgesia as previously shown; however, morphine analgesia was not affected by minocycline pretreatment in female rats. Minocycline decreased the expression of glial activation markers in the male spinal cord and periaqueductal gray as expected; however, these same molecules were upregulated in the female.

**Conclusions:**

These data describe a significant difference between males and females in the behavioral effects following co-administration of morphine and minocycline.

## Background

Sex differences in the brain have been studied for decades and are important for understanding sex differences in physiology and behavior. At baseline, sex differences in the brain can sometimes be small or insignificant but are often revealed or exacerbated following stress, injury, disease, or treatment with a drug. For example, opioids such as morphine are more effective at producing analgesia, or decreasing pain sensitivity, in males than in females [1–3]. In addition, male rats develop a greater tolerance to and dependency upon opiate drugs [4]. Increased opioid tolerance in males is associated with a significant *decrease* in the activation of neurons within the periaqueductal gray-rostral ventromedial medullary circuit compared to females in response to morphine [3], suggesting that sex differences in morphine function specifically within the periaqueductal gray (PAG) and its associated neural circuits have an important role in determining sex differences in morphine analgesia and tolerance [5].

Microglia are the primary immune cells of the brain. One of their functions is to detect and respond to infections, toxins, and physiological stressors within the central nervous system. One way in which they do this is via pattern recognition receptors (PRRs) located on their cell membrane. PRRs identify specific pathogen-associated molecular patterns (PAMPs) or more general “alarmins” (e.g., markers of cellular distress) that elicit the production of various cytokines and chemokines to stimulate a pro-inflammatory response and attract other immune cells to the affected area. For example, Toll-like receptor (TLR) 4 is a PRR which recognizes lipopolysaccharide (LPS), a cell wall component of the gram-negative bacteria. In addition to this classical function of TLR4, it also has the ability to respond, either directly or indirectly via endogenous alarmins/danger-associated molecular patterns (DAMPs), to a number of other foreign but non-pathogenic substances, including air pollution, alcohol, amphetamines, and opioids [6–10]. Specifically, morphine can activate the TLR4 receptor via its adapter protein, MD2, in addition to its ability to activate the classical opioid receptors (μ, δ, and κ opioid receptors) within the central nervous system [11]. Thus, drugs that inhibit the activity of microglia, such as minocycline, can enhance morphine’s analgesia and decrease the risk of tolerance, dependence, and associated reward [12–14]. Notably, this has only been previously explored in males. Thus, the current experiment sought to determine whether treatment with a microglial inhibitor, minocycline, could similarly enhance morphine analgesia in females, thereby potentially eliminating a sex difference in the efficacy of morphine.

Given the well-known sex difference in opioid analgesia and the recently discovered role of microglia in this aspect of opioid function, the purpose of this study was twofold. First, in Experiment 1, we determined whether inhibiting microglial activation using the tetracycline antibiotic minocycline would eliminate or significantly reduce the sex difference in morphine analgesia. Second, in Experiment 2, we examined whether treatment of males and females with a single acute dose of morphine produced a similar neuroimmune response within brain regions critical for opioid analgesia, including the ventrolateral (vl) PAG and the spinal cord and whether the expression of these immune molecules was similarly impacted by minocycline treatment in both males and females. Given that morphine analgesia is more effective in males than in females, we predicted that females would exhibit greater microglial activation to acute morphine administration than males and that minocycline would decrease this activation and thereby enhance morphine analgesia significantly more in females than in males.

Contrary to our initial predictions, pretreatment with minocycline exacerbated the sex difference observed in morphine analgesia (Experiment 1) and caused a differential neuroimmune response in males and females within the vlPAG and spinal cord (Experiment 2). Thus, in Experiment 3, we validated the effectiveness of the dose of minocycline used to inhibit the classic inflammatory response caused by activated microglia in these same brain regions critical for opioid analgesia.

## Methods

### Animals and drug

Sprague–Dawley rats from Harlan Laboratories, (Indianapolis, IN) were used for these experiments. They were housed in AAALAC-approved polypropylene cages on a 12:12-h light:dark cycle maintained at 22 °C with ad libitum access to food and water. All experiments were performed in accordance with the Institutional Animal Care and Use Committee of Duke University and the National Institutes of Health *Guide for the Care and Use of Laboratory Animals.* In Experiment 1, 16 male and 16 female rats were randomly assigned to one of two experimental groups: water + morphine (*n* = 8/sex) or minocycline + morphine (*n* = 8/sex) in order to test morphine analgesia on the hot plate. In Experiment 2, 20 male and 20 female rats were randomly assigned to one of four experimental groups: water + saline (*n* = 5/sex), water + morphine (*n* = 5/sex), minocycline + saline (*n* = 5/sex), or minocycline + morphine (*n* = 5/sex) for the analysis of glial (microglial and astrocytic) activation markers using real-time PCR.

Morphine sulfate was obtained courtesy of the National Institute of Drug Abuse (NIDA) Drug Supply Program and was dissolved in 0.9 % saline in the free-base concentrations described below. Minocycline hydrochloride (Cat. No. M9511) was obtained from Sigma Aldrich (St. Louis, MO) and dissolved in tap water for gavage at the concentrations described below.

### Experiment 1: determine the effect of minocycline on acute morphine-induced analgesia in male and female rats as measured on the hot plate

#### Time course of morphine analgesia on the hot plate

First, we examined the full-time course of morphine analgesia in males and females using the hot plate. Sixteen male and 16 female Sprague–Dawley rats were habituated for 1 min each day over 2 days to a hot plate while it was turned off. On the third day, the hot plate was set to 52.5 °C, and latency to respond to the heat was recorded when a rat picked up or licked its paw. Twenty minutes later, latency to respond to the hot plate was recorded again, and the average of these two latencies was calculated as the baseline. Following the baseline measurements, the rats were treated with a subcutaneous dose of morphine at either 4 mg/kg (*n* = 8/sex) or 8 mg/kg (*n* = 8/sex), and latency to respond to the hot plate was recorded 20, 40, 60, 90, and 120 min following the morphine injection. Throughout the course of all hot plate experiments, if the rat did not respond to the heat within 60 s, they were removed from the hot plate to avoid tissue damage, and they received a maximal response latency of 60 s.

The maximal percent effectiveness (%MPE) was determined by subtracting the average baseline response latency (baseline) from the response latency obtained after morphine administration and taking that as a percent of the maximal response latency (60-s cutoff) minus the average baseline response latency. Thus, for example, a maximum 60-s response latency would be 100 % MPE.

### Minocycline administration and %MPE analysis

After we determined the time course of %MPE of morphine on the hot plate, we used a separate cohort of rats to determine baseline latencies to respond to the hot plate following minocycline administration. Sixteen male and 16 female rats were habituated for 1 min each day over 3 days to a hot plate while it was turned off. At 8 pm on Day 3, half of the males (*n* = 8) and half of the females (*n* = 8) were gavaged with 50 mg/kg of minocycline, and the other half (*n* = 8/sex) were gavaged with an equal volume of tap water (1.25 ml/kg) as a control. Gavage was used in these experiments to administer minocycline because, despite the few seconds of stress associated with the procedure, it is a safe and highly effective route of administration for this drug, particularly versus intraperitoneal administration of minocycline which causes severe abdominal pain (unreported observations). In addition, the doses (50 mg/kg followed by 25 mg/kg 12 h later) and route of administration of minocycline were chosen for these experiments because they have previously been shown to effectively decrease levels of LPS-induced cytokine expression, particularly IL-1β in the male brain (Williamson et al. [15]). Ten hours after the first minocycline dose, the rats were placed on a hot plate set to 52.5 °C, and their latency to respond to the heat was recorded. Two baseline latencies were obtained from the rats at this time, approximately 20 min apart. The average of these two baseline latencies was calculated as Baseline 1.

Immediately after Baseline 1 was measured, rats were gavaged with the second dose of 25 mg/kg minocycline, and the control rats were gavaged with an equal volume of water (1.25 ml/kg). One hour after the second dose of minocycline was administered, the rats were tested two times, 20 min apart, for a second baseline latency to the hot plate set to 52.5 °C to determine whether the baseline latency to the heat changed after the immediate administration of minocycline (prior to the administration of morphine). The average of these two baseline latencies was calculated as Baseline 2. We saw no significant effect of treatment or sex on the baseline latency to respond to the hot plate before the morning dose of minocycline (data not shown).

Four hours after the second dose of minocycline was administered, all rats were injected subcutaneously with 1.8 mg/kg of morphine in 1 ml/kg saline. Twenty minutes later, all rats were tested for the latency to respond on the hot plate at 52.5 °C. Subsequent doses of 1.4, 2.4, 2.4, and 2 mg/kg of morphine were administered to the same rats after each test on the hot plate, a treatment regimen that results in quarter log serum doses of 1.8 mg/kg initially, then 3.2, 5.6, 8, and 10 mg/kg as previously published [5]. Again, throughout the course of the hot plate experiments, if the rat did not respond within 60 s to the hot plate, they were removed from the hot plate and received a maximal response latency of 60 s.

The maximal percent effectiveness (%MPE) was determined as described above. The maximum latency to respond to the hot plate was 60 s.

### Statistical analysis of maximal percent effectiveness

The %MPE at each dose was analyzed using a within subjects two-way ANOVA to analyze sex and minocycline as factors across doses. Significant interactions were followed up with Tukey’s post hoc tests (*p* < 0.05) to analyze individual comparisons.

#### Experiment 2: determine the effects of morphine and minocycline on pro-inflammatory gene expression and microglial activation in the spinal cord and periaqueductal gray of male and female rats

A second set of rats that did not undergo behavioral analysis on the hot plate was used for this experiment. Half of the rats in each sex (*n* = 10) were gavaged with 50 mg/kg of minocycline, and the other half (*n* = 10) were gavaged with an equal volume of tap water (1.25 ml/kg) as a control at 8 pm. The next day at 8 am, the same rats were gavaged with 25 mg/kg of minocycline or an equal volume of tap water (1.25 ml/kg). One hour after the second gavage, half of the rats in each treatment group (*n* = 5) were injected subcutaneously with either saline (1 ml/kg) or morphine (4 mg/kg) and sacrificed 20 min later. Four milligrams per kilogram of morphine was selected based on its striking sex difference in analgesic efficacy on the hot plate in Experiment 1.

#### Experiment 3: determine the effects of minocycline on expression of pro-inflammatory cytokine IL-1β following lipopolysaccharide treatment

An additional cohort of 32 male and 32 female Sprague–Dawley rats was used for this experiment. At 8 pm on Day 1, half of the rats in each sex (*n* = 16/sex) were gavaged with 50 mg/kg of minocycline, and the other half (*n* = 16/sex) were gavaged with an equal volume of tap water (1.25 ml/kg) as a control. Twelve hours later, at 8 am on Day 2, the same rats were gavaged with a second dose of minocycline at 25 mg/kg or an equal volume of tap water (1.25 ml/kg). One hour following the second gavage of minocycline or water, rats were given an intraperitoneal injection of either 100 μg/kg LPS at 1 ml/kg (*n* = 8/pretreatment/sex) or an equal volume of sterile saline (*n* = 8/pretreatment/sex). Four hours following either LPS or saline injection, the rats were euthanized, and the vlPAG, spinal cord, and hippocampus were collected for real-time PCR analysis of the classic pro-inflammatory cytokine interleukin-1β (IL-1β).

### Real-time PCR

The lumbar region of the spinal cord and micropunches of the ventrolateral (vl) periaqueductal gray (Bregma −7.0 mm to approximately −8.00, Experiments 2 and 3) and the whole hippocampus (Bregma −2.00 mm to approximately −7.00 mm, Experiment 3 only) were collected from each animal for the analysis of gene expression. mRNA was extracted from the microdissections using Isol-RNA Lysis Reagent (Cat. No. 2302700, 5 Prime, Gaithersburg, MD). Extracted RNA was subjected to DNase treatment and cDNA synthesis using the QuantiTect Reverse Transcription Kit and protocol from QIAGEN (Cat. No. 205311). Relative gene expression was measured using Bio-Rad Universal SYBR Green Supermix (Cat. No. 172–5121) in 10-μl reactions on a CFX96 Touch™ real-time PCR machine. The primers for each gene analyzed were ordered through Integrated DNA Technologies and diluted to a final concentration of 0.65 μM for the real-time PCR reaction. The sequences of the primers were as follows: GAPDH: forward: GTTTGTGATGGGTGTGAACC; reverse: TCTTCTGAGTGGCAGTGATG; GFAP: forward: AGGGACAATCTCACACAGG; reverse: GACTCAACCTTCCTCTCCA; CD11b: forward: CTGGGAGATGTGAATGGAG; reverse: ACTGATGCTGGCTACTGATG; Toll-like receptor (TLR) 4 forward: CAGAGGAAGAACAAGAAGC; reverse: CCAGATGAACTGTAGCATTC; CX_3_CR_1_: forward: TTCCTCTTCTGGACGCCTTA; reverse: TAAACGCCACTGTCTCCG; CX_3_CL_1_: forward: TCCAGGGCTGTCCCCGCAAA; reverse: ACAGGCAGGCAAGCAGGCAG; and IL-1β: forward: GAAGTCAAGACCAAAGTGG; reverse: TGAAGTCAACTATGTCCCG.

### Real-time PCR analysis

For each sample’s reaction, the quantitative threshold amplification cycle number (*C*_*q*_) was determined, and the 2^−ΔΔCq^ method was used using GAPDH as the reference gene to calculate the relative gene expression.

### Statistical analysis of gene expression

For each gene, a 2 × 2 × 2 ANOVA with sex, minocycline, and morphine (Experiment 2) or LPS (Experiment 3) as variables was used to analyze the relative gene expression and determine significant (*p* < 0.05) interactions of sex, morphine, and/or minocycline/LPS and significant main effects of minocycline and morphine/LPS. Only significant interactions were followed up with Tukey’s post hoc test (*p* < 0.05) to analyze individual comparisons.

### Estrous cycle analysis

For most experiments, vaginal swabs were taken from females to determine estrous cycle at the time of behavioral analysis or tissue collection, and based on our analysis, cycle phase did not significantly explain variability within or sex differences observed between treatment groups.

## Results

### Experiment 1: examine the effect of minocycline on morphine-induced analgesia in male and female rats

#### Time course analysis of morphine analgesia in males and females

**Fig. 1:**
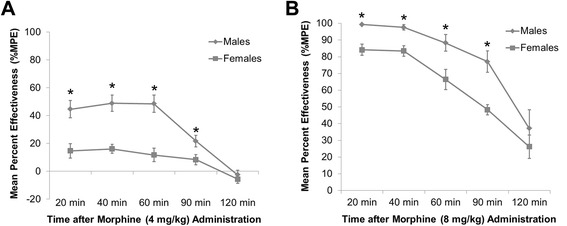
Mean percent effectiveness of acute morphine analgesia in male and female rats. Male and female rats were treated with either 4 mg/kg or 8 mg/kg of morphine sulfate subcutaneously. Morphine analgesia was measured using a hot plate set at 52.5 °C. The latency to respond to the heat with a rear paw shake or lick was recorded 20, 40, 60, 90, and 120 min post morphine administration. **a** Morphine produced significantly greater analgesia in males than females at a dose of 4 mg/kg (repeated-measures ANOVA: *asterisk* represents main effect of sex: *F*_1,15_ = 21.22; *p* = 0.00040). **b** Morphine produced significantly greater analgesia in males than females at a dose of 8 mg/kg (repeated-measures ANOVA: *asterisk* represents main effect of sex: *F*_1,15_ = 49.95; *p* < 0.001)

We found a significant sex difference in morphine analgesia such that both the 4 mg/kg and the 8 mg/kg doses of morphine produced significantly greater analgesia on the hot plate in males compared to females (repeated-measures ANOVA: main effect of sex: *F*_1,15_ = 21.22; *p* = 0.00040, Fig. 1a**,** and *F*_1,15_ = 49.95; *p* < 0.001, Fig. 1b, respectively). Given that morphine analgesia peaked 20 min post subcutaneous morphine administration and that the sex difference was present 20 min post morphine administration, we chose 20 min as our time point of analysis for our subsequent experiments.

#### Baseline hot plate latencies

We found no significant main effect or interaction of sex or minocycline treatment on the baseline latencies obtained on the hot plate at any time point examined (Baseline 1: sex - *F*_1,31_ = 1.63, *p* = 0.21; minocycline - F_1,31_ = 0.03, *p* = 0.86; sex × minocycline - *F*_1,31_ = 0.71, *p* = 0.40; Baseline 2: *F*_1,31_ = 1.18, *p* = 0.28; minocycline - *F*_1,31_ = 0.07, *p* = 0.79; sex × minocycline - *F*_1,31_ = 0.01, *p* = 0.92). Baseline latencies to respond on the hotplate were as follows (mean ± SEM) [females + water: 12.5 ± 0.73 s, females + minocycline: 11.5 ± 1.22 s, males + water: 10.5 ± 0.87 s, males + minocycline: 11.15 ± 0.94 s] or after the morning dose of minocycline [females + water: 13.81 ± 1.85, females + minocycline: 13.58 ± 1.50 s, males + water: 12.36 ± 1.31 s, males + minocycline: 11.8 ± 1.20 s]. These data indicate that neither sex nor minocycline treatment affected the baseline latency to respond on the hot plate.

#### Morphine analgesia with or without minocycline

**Fig. 2:**
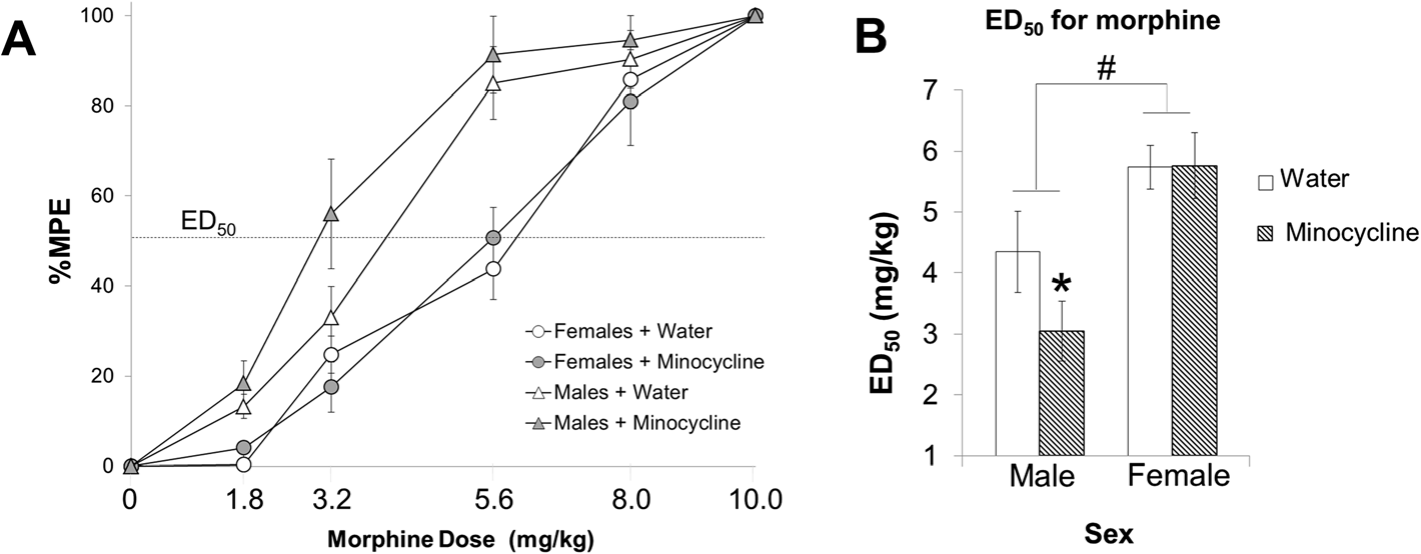
Effects of minocycline and dose responses to morphine on the hot plate. After habituation to the apparatus, *n* = 8 rats per sex were gavaged with 50 mg/kg minocycline, and *n* = 8 rats per sex were gavaged with an equal volume of water (1.25 ml/kg). Their latencies to respond to the painful stimulus of the hot plate were taken 10 h post gavage, and they were recorded twice 20 min apart and averaged as Baseline 1 (see “Methods” section for full details). Immediately following Baseline 1 measurements, rats were either gavaged with a second dose of minocycline (25 mg/kg) or equal volume of water a second time (1.25 ml/kg). One hour after the second gavage, latencies to respond to the stimulus on the hot plate were recorded twice and averaged to obtain Baseline 2 (**a**). The dose response curve following incremental doses of morphine (mg/kg) administration is graphed for rats in all groups. ED_50_ is marked to represent the doses of morphine that elicit 50 % MPE (**b**). The ED_50_ (mg/kg) doses of morphine for males and females treated with or without minocycline are graphed to show the values of morphine required to obtain 50 % MPE. *Asterisk* represents significant interaction of sex and minocycline treatment (*p* < 0.05); *number sign* represents main effect of sex (*p* < 0.001)

Figure 2a depicts the dose response curve obtained following incremental doses of morphine administration in males and females treated with or without minocycline. Similar to previous reports, we found a significant main effect of sex on the analgesic response to morphine such that males experience greater analgesia on the hot plate at lower doses of morphine (main effect of sex: *F*_1,26_ = 37.01; *p* < 0.001). Interestingly, we also found a significant interaction of sex and minocycline treatment across doses (sex × treatment: *F*_1,26_ = 4.56; *p* = 0.041). Post hoc analysis revealed that minocycline significantly shifted the dose response curve only in males such that males treated with minocycline exhibited significantly greater analgesia to morphine at lower doses of morphine than males treated with water (*p* < 0.05). In contrast, post hoc comparisons revealed that minocycline had no effect in females compared to controls (*p* = 0.82). We also calculated the ED_50_, or the dose at which morphine produced a mean percent effectiveness of 50 % (MPE 50 %) for morphine on the hot plate, for the each rat (Fig. 2b). As expected, the statistical analysis was identical to the repeated measures above and revealed a significant main effect of sex (*F*_1,26_ = 37.01; *p* < 0.001) and a significant interaction of sex and minocycline treatment (*F*_1,26_ = 4.56; *p* = 0.041) on the ED_50_.

### Experiment 2: determine the effects of morphine and minocycline on pro-inflammatory gene expression and microglial activation in the spinal cord and periaqueductal gray of male and female rats

#### Glial activation markers and immune molecules in the spinal cord

**Fig. 3:**
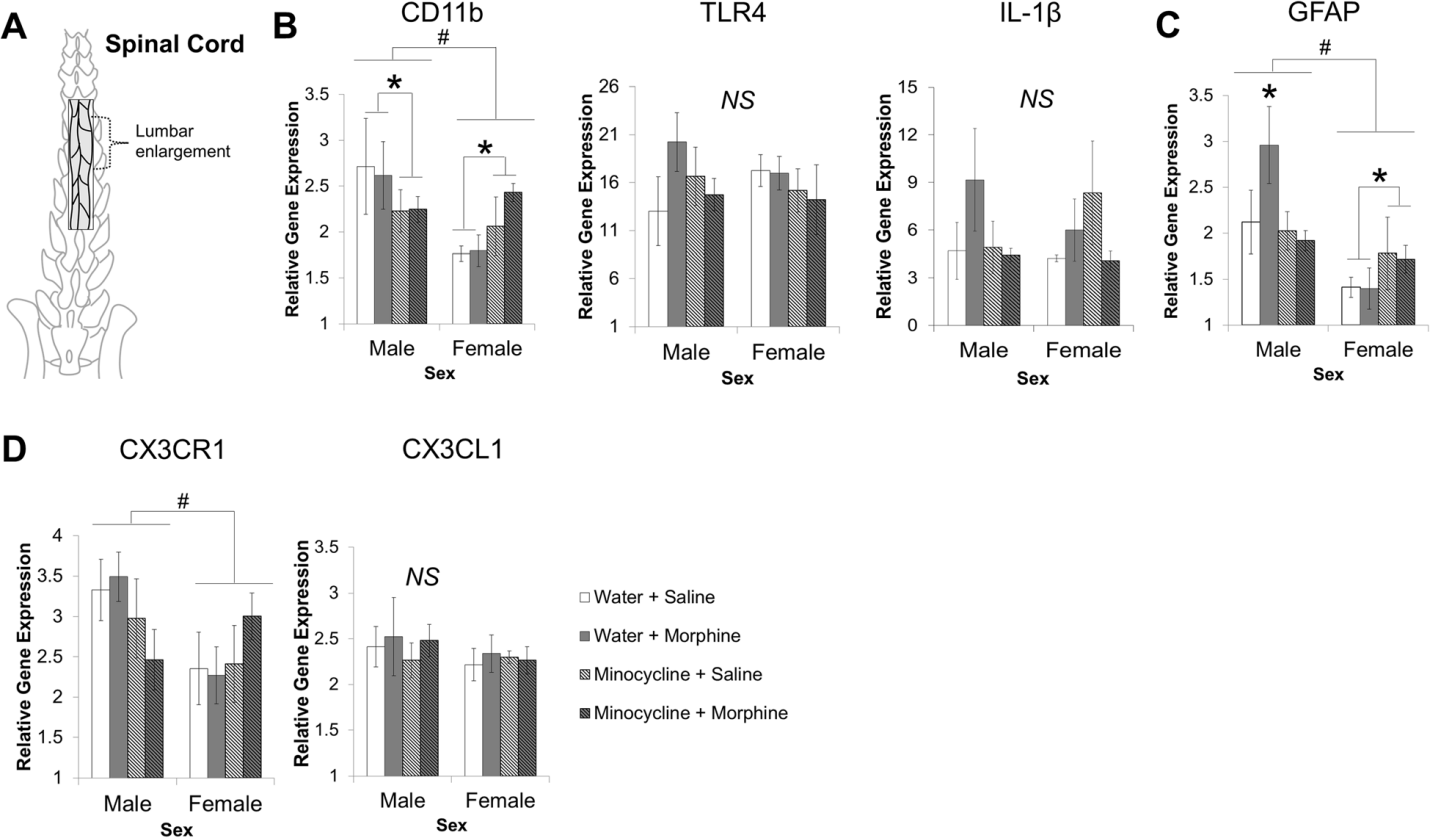
Relative gene expression in the lumbar region of the spinal cord. **a** depicts a diagram of the lumbar enlargement that was taken for RNA extraction, cDNA synthesis, and real-time PCR analysis. Males and females were treated with either water (*n* = 8 per sex) or minocycline (*n* = 8 per sex; 50 and 25 mg/kg 12 h later) then treated with morphine (4 mg/kg) or saline s.c. Tissue was collected 20 min post morphine administration. Data were analyzed using a 2 × 2 × 2 ANOVA with sex, morphine treatment, and minocycline treatment as variables. Main effects and interactions are depicted as indicated below. Graphs represent the mean ± SEM. **b** depicts the analysis of microglial and inflammatory markers of the spinal cord, specifically CD11b, TLR4, and IL-1β. Analysis of CD11b revealed a main effect of sex (^#^ *p* < 0.05) and interaction between sex and minocycline treatment (*p < 0.05). **c** Analysis of the astrocyte marker, GFAP, shows a significant interaction between sex and minocycline treatment (*p < 0.05) and a main effect of sex (^#^ *p* = 0.001). **d** depicts the analysis of the fractalkine receptor CX3CR1 and its ligand CX3CL1. CX3CR1 reveals a main effect of sex (^#^ *p* < 0.05)

First, we analyzed the lumbar enlargement of the spinal cord (Fig. 3a) for microglial and astrocytic activation markers that have previously been shown to be increased in response to morphine treatment within the brain (Schwarz et al. [16]; [17]). Given our interesting behavioral results, we hypothesized that morphine treatment might activate microglia in the spinal cord to a greater extent in females than males and that minocycline treatment would only be effective at reducing microglial activation in males and not in females.

Analysis of CD11b, a marker of microglial activation, revealed a significant interaction of sex and minocycline treatment (*F*_1,32_ = 5.15, *p* = 0.03), such that minocycline significantly inhibited CD11b expression in males (*p* < 0.05) but significantly increased CD11b expression in females (*p* < 0.05; Fig. 3b). Surprisingly, however, we found no effect of morphine on CD11b expression in either sex. We also analyzed the expression of TLR4, the innate immune receptor expressed on the surface of microglia that responds to morphine exposure [11], to determine whether females have significantly more TLR4 expression in the spinal cord than males. Statistical analysis did not reveal any significant main effects or interactions of sex or treatment on TLR4 expression in the spinal cord (Fig. 3b). Next, we examined the classic proinflammatory cytokine IL-1β; however, we did not find any significant main effects or interactions of sex, minocycline, or morphine in the spinal cord (Fig. 3b).

GFAP is an astrocytic marker that also increases expression in response to morphine administration in the brain (Schwarz et al. [16]). We sought to test the alternate hypothesis that morphine could activate astrocytes in the spinal cord to a greater extent in females than in males and thus inhibit morphine analgesia significantly more in females than in males. In addition, because astrocytes are not a known target for minocycline, this alternate hypothesis could also explain the lack of effect of minocycline in females relative to males. Interestingly, statistical analysis of GFAP in the spinal cord revealed a significant interaction between sex and minocycline treatment (*F*_1,32_ = 5.59, *p* = 0.024) such that minocycline significantly inhibited a morphine-induced increase in GFAP expression in the male spinal cord (*p* < 0.05), but in contrast minocycline significantly increased GFAP expression in females (*p* < 0.05; Fig. 3c).

Next, we examined the expression of the fractalkine receptor (CX3CR1), a chemokine receptor expressed by microglia, and its ligand, CX3CL1, a chemokine produced by neurons that modulates microglial activation via activation of CX3CR1. Based on previous reports (Schwarz et al. [16]; [18]), we hypothesized that if morphine activates neurons via its classical opioid receptor pathways that neurons may, in turn, decrease CX3CL1 production, which might increase microglial activation; and this mechanism may be significantly different between males and females and/or significantly inhibited by minocycline treatment. Statistical analysis of the fractalkine receptor (CX3CR1) revealed a significant main effect of sex (*F*_1,32_ = 3.97, *p* ≤ 0.05; Fig. 3d), as males had significantly elevated levels of CX3CR1 in the spinal cord relative to females. The expression of the chemokine, CX3CL1, did not show any main effects or interactions of sex or treatment (Fig. 3d).

#### Glial activation markers and immune molecules in the ventrolateral periaqueductal gray

**Fig. 4:**
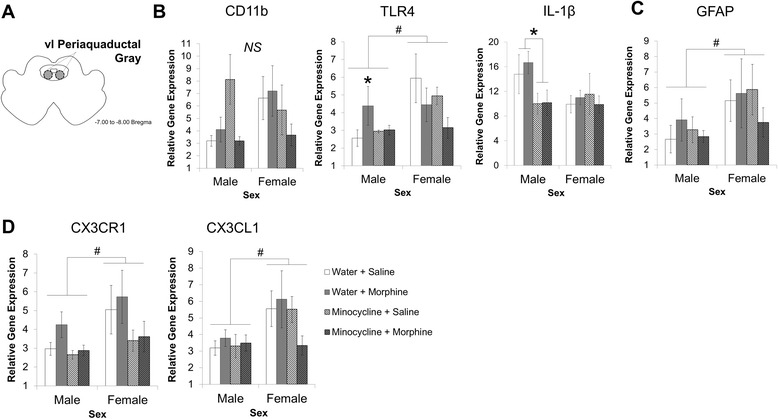
Relative gene expression in the ventrolateral periaqueductal gray (vlPAG). **a** represents a diagram of the ventrolateral periaqueductal gray region that was taken for RNA extraction, cDNA synthesis, and real-time PCR analysis. Males and females were treated with either water (*n* = 8 per sex) or minocycline (*n* = 8 per sex; 50 and 25 mg/kg 12 h later) then treated with morphine (4 mg/kg) or saline s.c. Tissue was collected 20 min post morphine administration. Data were analyzed using a 2 × 2 × 2 ANOVA with sex, morphine treatment, and minocycline treatment as variables. Main effects and interactions are depicted as indicated below. Graphs represent the mean ± SEM. **b** shows the graph of relative gene expression analysis using GFAP. *Number sign* represents a main effect of sex (*p* < 0.05). **c** depicts the gene analysis for CD11b, TLR4, and IL-β in the vlPAG. TLR4 shows a main effect of sex (^#^ *p* < 0.05) and an interaction between sex and minocycline treatment (*p < 0.05). **d** represents the relative gene expression analysis of fractalkine receptor CX3CR1 and its ligand CX3CL1. Both show a main effect of sex (^#^ *p* < 0.05)

Next, we examined the expression of these same genes in the ventrolateral periaqueductal gray (vlPAG, Fig. 4a) with the same predictions in mind; that morphine treatment might activate microglia in the vlPAG to a greater extent in females than in males and that minocycline treatment would only be effective at reducing microglial activation in males and not in females.

We found no significant effect of sex or treatment on CD11b expression in the vlPAG (Fig. 4b). However, statistical analysis of TLR4 expression in the vlPAG revealed a significant interaction of sex and morphine (*F*_1,32_ = 5.52, *p* = 0.02; Fig. 4b). Specifically, morphine increased TLR4 expression in the vlPAG of males (*p* < 0.05) and this effect was significantly inhibited by pretreatment with minocycline (*p* < 0.05). In contrast, females had significantly elevated levels of TLR4 relative to males and these levels were not significantly influenced by morphine treatment and/or minocycline administration. Next, IL-1β expression showed a trending interaction of sex and minocycline (*F*_1,32_ = 3.78, *p* = 0.061; Fig. 4b). Following these data, we ran separate 2 × 2 ANOVA tests within the males and females and found a significant main effect of minocycline treatment in only the males (*F*_1,32_ = 6.28, *p* = 0.0234) such that males that were administered minocycline had significantly lower expression of IL-1β compared to their water-treated controls.

There was a significant main effect of sex on GFAP expression in the vlPAG (*F*_1,32_ = 4.31, *p* = 0.04, Fig. 4c), such that females had significantly more GFAP expression in the vlPAG than males regardless of treatment. No other effects or interactions were determined to be statistically significant.

We also measured the relative gene expression for the chemokine receptor (CX3CR1) and its ligand (CX3CL1) in the vlPAG. In this region, the receptor (CX3CR1) showed a significant main effect of sex (*F*_1,32_ = 4.78, *p* = 0.03) and of minocycline (*F*_1,32_ = 5.54, *p* = 0.02). The cytokine, CX3CL1 (fractalkine) also showed this main effect of sex (*F*_1,32_ = 7.37, *p* = 0.01) but showed no other significant results (Fig. 4d).

### Experiment 3: determine the effects of minocycline on expression of pro-inflammatory cytokine IL-1β following lipopolysaccharide treatment

Given our unexpected findings from Experiments 1 and 2, we sought to confirm the dose and effectiveness of minocycline as a drug used to inhibit inflammation as caused by activated microglia in both males and females. Thus, we treated males and females with or without the same dose of minocycline used in the previous experiments, followed by administration of LPS or saline.

**Fig. 5:**
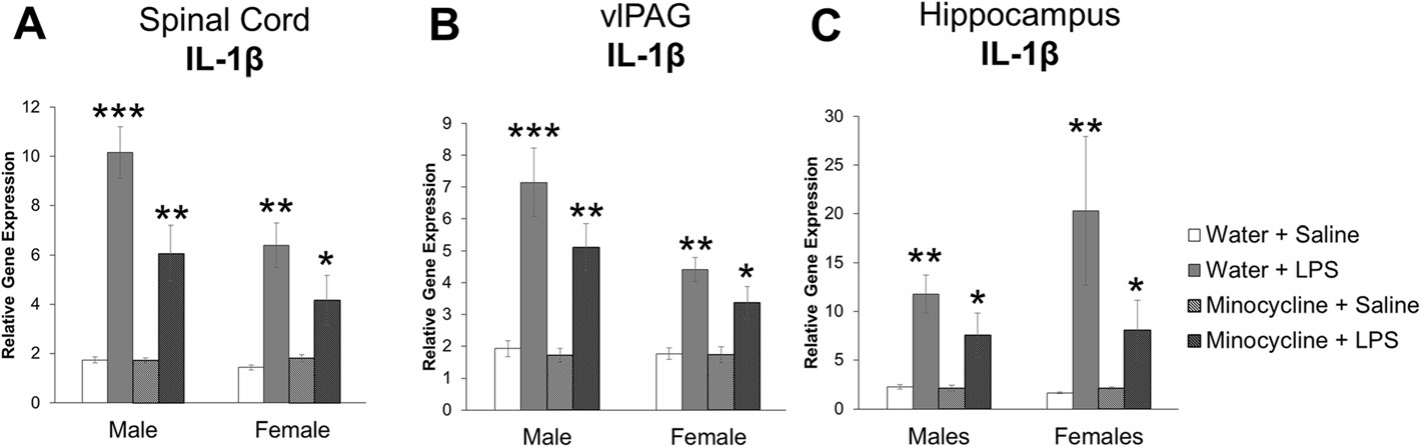
Relative gene expression of IL-1β mRNA in the spinal cord, ventrolateral periaqueductal gray, and hippocampus of male and female rats treated with lipopolysaccharide (100 μg/kg). Male and female rats were treated with either minocycline (one 50 mg/kg dose and second 25 mg/kg dose administered 12 h apart) or water and 1 h after that with either 100 μg/kg of lipopolysaccharide (LPS) or sterile saline (1 ml/kg) intraperitoneal (*n* = 8/group). Four hours later, the spinal cord, ventrolateral periaqueductal gray (vlPAG), and hippocampus were dissected for analysis of IL-1β mRNA expression. Graphs represent the mean ± SEM. Different asterisk combinations (e.g., * vs. ** vs. ***) represent significantly different groups. Thus, similar asterisk combinations are not significantly different from each other. **a** LPS significantly increased the production of IL-1β in the spinal cord of both males and females but to a greater extent in males than in females (sex × LPS interaction: *F*_1,62_ = 7.48; *p* = 0.008). Minocycline significantly attenuated the increase in LPS in both males and females (minocycline × LPS interaction: *F*_1,62_ = 11.21; *p* = 0.001). We found no significant interaction of minocycline and sex (*p* = 0.263). **b** We found similar effects of LPS and minocycline treatment on IL-1β expression in the vlPAG (sex × LPS interaction: *F*_1,62_ = 8.17; *p* = 0.006; minocycline × LPS interaction: *F*_1,62_ = 4.63; *p* = 0.037). **c** LPS significantly and similarly increased IL-1β in the hippocampus of both males and females (main effect of LPS: *F*_1,62_ = 18.47; *p* < 0.001; minocycline × LPS: *F*_1,62_ = 3.32; *p* = 0.072)

Analysis of IL-1β in the lumbar enlargement of the spinal cord revealed an interaction of sex and LPS such that IL-1β expression was significantly more elevated in males after LPS treatment compared to females (*F*_1,61_ = 7.487; *p* = 0.008, Fig. 5a). Analysis also revealed an interaction of minocycline and LPS such that minocycline pretreatment inhibited the LPS-induced increase in IL-1β expression in both males and females (*F*_1,61_ = 11.213, *p* = 0.001, Fig. 5a), and there was no significant interaction of sex and minocycline treatment.

We found similar effects in the analysis of IL-1β in the vlPAG such that there was an interaction of sex and LPS with males having a greater increase in IL-1β after LPS administration compared to females (*F*_1,61_ = 8.178, p = 0.006) and an interaction of minocycline and LPS such that minocycline inhibited the LPS-induced increase in IL-1β expression in both males and females (*F*_1,61_ = 4.632, *p* = 0.37, Fig. 5b), with no significant interaction of sex and minocycline treatment.

Lastly, we analyzed the expression of IL-1β in the hippocampus of these rats as the hippocampus is particularly sensitive to immune activation. Analysis revealed a main effect of LPS treatment such that in both males and females, LPS significantly increased the expression of IL-1β (*F*_1,61_ = 18.427, *p* < 0.001, Fig. 5c). Additionally, we found a trend towards an interaction of minocycline and LPS such that minocycline pretreatment inhibited the LPS-induced increase of IL-1β expression (F_1,61_ = 3.372, p = 0.072, Fig. 5c).

## Discussion

The current experiments were designed to test whether minocycline could enhance morphine analgesia in both males and females, thereby eliminating the well-known sex difference in morphine analgesia [2, 19–24]. Previous reports indicate that morphine can activate microglia and astrocytes in the brain and spinal cord and that associated inflammation or TLR4 activation can inhibit or counteract the analgesic properties of morphine [12, 25, 26]. These non-specific effects of morphine treatment can be reversed using the microglial inhibitor, minocycline [27, 28]; however, this effect had only been previously examined in males despite well-known differences in immune function and morphine analgesia between males and females. Thus, this study sought to determine whether minocycline treatment could increase the efficacy of morphine in both males and females and thereby potentially eliminate or reduce the sex difference in the efficacy of this important opiate drug. First, our results confirmed the numerous reports of sex differences in morphine analgesia. Second, our data revealed an even more striking sex difference in the efficacy of the microglial inhibitor, minocycline, in its ability to impact morphine analgesia in males but not in females. Finally, a preliminary investigation of microglial activation markers and associated immune molecules in the spinal cord and vlPAG uncovered additional sex differences in the effect of minocycline on these immune molecules that may reveal potential mechanisms contributing to the sex difference in morphine analgesia and minocycline efficacy.

As expected, males exhibited greater morphine-induced analgesia on the hot plate relative to females, data that corroborates an abundance of previous literature [1–3, 19–24] demonstrating this significant sex difference in morphine effectiveness. Previous research indicates that glial inhibitors, such as minocycline, significantly enhance morphine-induced analgesia in males [27, 28], suggesting that in the absence of minocycline, morphine activates microglia and inhibits its analgesic properties even after acute morphine administration. Thus, we examined the impact of morphine and minocycline on morphine analgesia and the activation of microglia and astrocytes 20 min after morphine administration. We hypothesized that morphine would activate microglia to a greater extent in females than in males and thus counteract the analgesic properties of morphine significantly more in females compared to males. Minocycline treatment significantly increased the analgesic efficacy of morphine in males as previously reported, but surprisingly, minocycline did not affect morphine-induced analgesia at all in female rats. The dose response curves in Fig. 2a depict a significant leftward shift in the curve for males that were treated with minocycline, but this same shift is not seen in the dose response curve of females treated with minocycline.

These results did not support our initial hypothesis and would suggest one of three things: either (1) the males have significantly more morphine-induced microglial activation than females within brain regions that modulate morphine-induced analgesia and thus inhibiting microglial activation with minocycline enhances morphine-induced analgesia significantly more in males than it does in females; or (2) this dose of minocycline (one 50 mg/kg dose and second 25 mg/kg dose administered 12 h apart) is not as effective at inhibiting morphine-induced microglial activation in females as it is in males, regardless of the relative levels of morphine-induced microglial activation; or (3) while microglia may influence morphine analgesia in males, this is not the case in females, and in fact microglia have effect on morphine analgesia in females. To further investigate these potential mechanisms, the expression of the microglial activation marker, CD11b, and other immune molecules in the lumbar enlargement of the spinal cord and the vlPAG were examined.

The expression pattern for a number of genes in both the spinal cord and the vlPAG were consistent with behavioral findings, though a few were not. Surprisingly, CD11b expression, though often used as a proxy of microglial activation, was not increased following morphine administration in either the spinal cord or the vlPAG of either sex. Given the evidence, it appears that CD11b is not an appropriate marker for microglial activation in this particular model of acute morphine administration, and that microglia may in fact be “active” within 20 min post morphine administration but that CD11b is not simultaneously upregulated as a result. Additionally, morphine had no effect on the expression of the classic pro-inflammatory marker IL-1β in either the spinal cord or vlPAG of males and females. In contrast, morphine did increase the expression of TLR4 in the vlPAG and of GFAP in the spinal cord after only 20 min, though only in male rats. This sex difference in the response to morphine for TLR4 and GFAP supports the first of the three potential explanations proposed above, that males may in fact have greater morphine-induced activation of glia such that inhibition of glia with minocycline produces an increase in morphine analgesia selectively in males. However, it is also worth noting the significant sex difference in baseline TLR4 expression in the vlPAG where control females have significantly more TLR4 than control males. This might suggest that females are more sensitive to PAMPs compared to males. Contrastingly, in our subsequent experiment using minocycline and LPS administration, we see that LPS induces IL-1β expression significantly more in males compared to females. Thus, it is possible that glial activation by either morphine or LPS administration causes males to make more TLR4 in response, as seen 20 min following morphine administration. These data support our third hypothesis that suggests microglia influence male morphine analgesia and do not influence female morphine analgesia, especially given that we do not see a similar increase in TLR4 expression in females following morphine administration at this time.

We also hypothesized that the function of the fractalkine receptor, CX3CR1, and its chemokine ligand, CX3CL1, would impact morphine analgesia and may be subsequently affected by minocycline pre-treatment. Previous literature supports an anti-nociceptive function of the fractalkine receptor in that that binding the ligand CX3CL1 produced by neurons to the microglial receptor CX3CR1 inhibits microglial function [29, 30]. We predicted that if morphine activated neurons through its classic opioid receptor pathway, neurons would decrease the production of the ligand and thus increase microglial activation and prevent anti-nociception [18]. While we do not find any significant effects of morphine on the expression of CX3CL1 in either the spinal cord or PAG of either sex, we do see significant differential sex differences in the expression of the receptor CX3CR1 in the spinal cord and PAG. Furthermore, other bodies of literature describe a pro-nociceptive function of fractalkine in the spinal cord [31, 32]. Given these conflicting evidence and findings, it is difficult to interpret the function of this sex difference in the fractalkine receptor on morphine analgesia at this time.

Although morphine did not increase CD11b in the spinal cord or vlPAG of either sex, minocycline did alter the expression of immune markers in both the spinal cord and vlPAG of males and females, though not entirely as predicted. In support of our initial hypothesis and the behavioral data, minocycline treatment inhibited CD11b expression in the male spinal cord. Additionally, expression of IL-1β was significantly decreased as a result of minocycline but in only male rats. Minocycline also significantly inhibited the morphine-induced upregulation of GFAP expression in the spinal cord of male rats and the morphine-induced upregulation of TLR4 in the vlPAG. The significant decrease in GFAP expression in males was quite intriguing as we did not expect minocycline to have such an effect on astrocytes. However, other reports indicate that minocycline can inhibit the activation of astrocytes in addition to microglia [33–35] and given the significant interactions between microglia and other cells in the brain, including astrocytes, it is possible that by inhibiting microglial activation with minocycline, we indirectly impact the function of astrocytes and the expression of GFAP.

In contrast, minocycline increased CD11b and GFAP expression in the spinal cord of females, data that is consistent with the behavioral findings presented here indicating that minocycline does not enhance morphine-induced analgesia in females. It is not clear why minocycline would have such opposing effects on the physiology of males and females; however, sex differences in the physiological effects of minocycline are not unique to the present study. For example, in a model of ischemic stroke, minocycline (two 45 mg/kg doses administered 12 h apart) successfully inhibited the stroke-induced upregulation of poly (ADP-ribose) polymerase, a DNA repair enzyme associated with cell death, in *both* males and females, yet minocycline was only neuroprotective against this model in *male* mice [36]. Additionally, our data indicate that minocycline increases the expression of microglial and astrocyte activation markers in the spinal cord and vlPAG of only female rats, which suggests that this dose does have a physiological effect, though not the same effect produced in males.

It is also possible that the acute pain produced by exposure to the hot plate in these experiments initiates a differential inflammatory or cellular immune response in males and females such that treatment with minocycline inhibits the pain-induced activation of microglia in males, thereby enhancing analgesia independent of morphine, while minocycline has no effect on pain-induced activation of microglia in females, thereby having no effect on morphine analgesia as our third hypothesis states. This working hypothesis is supported by a recent report indicating that pain or nerve injury activates microglia in males, while the same pain or nerve injury in females activates and recruits T cells. In this study, inhibiting microglia had no impact on the pain hypersensitivity produced by nerve injury in females, but it significantly decreased pain hypersensitivity to the same injury in males [37]. However, additional studies have used varying doses of minocycline and found successful suppression of inflammatory pain [38] and neuroprotection following thromboembolitic stroke [39] in both males and females. In addition, our data also indicate that the current dose of minocycline inhibits the synthesis of IL-1β stimulated by lipopolysaccharide exposure to similar levels in both males and females (Fig. 5). In conclusion, this literature and the data presented here indicate that the sex difference observed in minocycline efficacy may be the result of sex differences in the mechanism of action of minocycline subsequently *interacting with* the mechanism of the disease (or in this case the other drug, morphine) and is not a result of sex differences in the required dose for efficacy.

Also in support of this hypothesis, it is well known that there are other factors that can influence the analgesic properties of morphine in females. For example, sex differences in mu opioid receptor number and sensitivity contribute to the well-known sex difference in opioid analgesia, tolerance, and dependence [3, 5, 40]. In addition, hormonal fluctuations associated with the estrous cycle contribute to differential sensitivity of morphine in females as these hormones mediate the expression and function of opioid receptors in the female spinal cord [41–46]. These hormones also inhibit anti-nociception by morphine at the level of the vlPAG in females but not males [47–51], and these circulating hormones impact the function of neurons in hypothalamic nuclei that directly interact with these downstream targets [47]. It is also possible that hormones may similarly influence the glial or neuroimmune responses to morphine in female rats independent of minocycline. In one study, TLR4 activation via LPS produced allodynia in only male rats [52]. Researchers found that removing male circulating testosterone significantly alleviated LPS-induced allodynia, suggesting that testosterone mediated the TLR4 signaling pathway in male rats while this affect is not seen in female rats. Additionally, it has been demonstrated that female sex steroids also influence the neuroimmune response to LPS. LPS-induced pro-inflammation in male rats was significantly attenuated in the presence of estradiol while pro-inflammation in female rats was potentiated (Loram et al. [53]). Though we found no impact of estrous cycle phase on the behavioral or cellular response to morphine in females in Experiments 1 and 2, our findings do not discount the additional role that the estrous cycle phase may have on any of the cellular effects of morphine described above.

## Conclusions

Most importantly, these data indicate that the effects of drugs must be examined in both males and females. We and others have repeatedly demonstrated that opioids are more effective analgesics in males than in females. Here, we also found that minocycline was significantly more effective at inhibiting both microglia and astrocyte activation in males than in females. In fact, in females, this dose of minocycline appears to increase microglial activation, though we see no decrease in morphine effectiveness in the hot plate following minocycline treatment. These data exemplify the growing consensus in biomedical research that sex must be considered as a factor when examining the effectiveness, potential side effects, and interactions of every medication. In this case, the combined treatment of morphine and minocycline exaggerated the baseline sex difference in the effectiveness of one drug and resulted in significant differences in cell-type specific function in the brain and spinal cord. Thus, our findings extend the literature on sex differences in morphine analgesia and elaborate on potential mechanisms for this sex difference. These data highlight the importance of examining both sex and neural cell type when considering the interactions, physiological effects, and behavioral effects of specific therapeutic drugs alone or in combination.
